# Is a Pacific Coexistence Between Virus and Host the Unexploited Path That May Lead to an HIV Functional Cure?

**DOI:** 10.3390/v5020753

**Published:** 2013-02-21

**Authors:** Jonathan Fior

**Affiliations:** Immunobiology of HIV Unit, Division of Immunology, Transplantation and Infectious Diseases, San Raffaele Scientific Institute, via Stamira D’Ancona 20, 20127 Milan, Italy; E-Mail: jonathan.fior82@gmail.com

**Keywords:** HIV infection, Functional cure, Cell-based therapy, Vif, APOBEC3G, SupT1 cells, HIV reservoir, CD4+ T cells

## Abstract

The SupT1 cell line supports optimal HIV-1 replication, and prolonged *in vitro* replication in SupT1 cells renders the virus significantly less virulent. This raises the question of whether the infusion of SupT1 cells could be used as a cell-based therapy to induce a pacific coexistence between the HIV virus and its human host. In a recent study, I investigated this potential therapeutic strategy *in vitro*. The results suggested that this approach should be further explored in HIV-susceptible animal models. Such studies may lead to the development of a functional cure for HIV infection.

## 1. Introduction

The course of HIV-1 infection in humans is usually characterized by a progressive increase in viral pathogenicity, which leads to the development of AIDS. Along with CD4+ T cell depletion, another pathological hallmark of HIV-1 infection is chronic immune activation [1]. By contrast, natural SIV hosts such as African green monkeys and sooty mangabeys do not develop AIDS, although they harbor high SIV viral loads. Furthermore, SIV infection does not trigger chronic immune activation in these animal hosts [2]. A possible explanation for the progressive increase in viral pathogenicity during the course of HIV-1 infection is that the cells of the host do not support optimal viral replication; therefore, the selection of more virulent HIV-1 variants is the evolutionary route chosen by the virus. 

Interestingly, the SupT1 cell line supports optimal HIV-1 replication, and prolonged *in vitro* replication in SupT1 cells renders the virus significantly less virulent. To be more specific, *in vitro* studies of HIV evolution show that persistent growth in the SupT1 cell line results in a less cytopathic virus with a reduced capacity for syncytium formation, a higher sensitivity to neutralization, improved replication in SupT1 cells and impaired infection of primary CD4+ T cells [3,4,5]. To date, the therapeutic potential of this phenomenon remains largely unexplored. These observations raise the question of whether SupT1 cell infusions could form the foundation of a cell-based therapy that has the potential to induce a pacific coexistence between the HIV virus and its human host.

## 2. New Findings and Discussion

In a recent *in vitro* study [6], I investigated the strategy of using inoculated SupT1 cells to move HIV infection toward the inoculated cells, which should theoretically prevent infection and depletion of normal CD4+ T cells, preventing the development of AIDS-related pathologies. I used infected SupT1/PBMC co-cultures and a series of control experiments to mimic a situation in which SupT1 cells are inoculated into an HIV-seropositive patient. Infections were performed using the highly pathogenic wild-type HIV-1 LAI virus. The results showed that the virus-mediated killing of primary CD4+ T cells was significantly delayed in the SupT1/PBMC co-cultures, suggesting that the preferential infection of SupT1 cells can spare primary CD4+ T cells from infection and depletion [6]. Interestingly, as previously mentioned, *in vitro* studies of HIV evolution show that prolonged replication in SupT1 cells renders the virus less cytopathic and more sensitive to neutralization [3,4,5], suggesting that replication of the virus in the inoculated SupT1 cells may also have a vaccination effect, in the long run. Thus, further exploration of the SupT1 cell line as a cell-based therapy for HIV may prove worthwhile. In this respect, it is interesting that the HIV-1 accessory protein Vif is essential for viral replication in primary CD4+ T cells but not in SupT1 cells [7]. Therefore, a drug that specifically inhibits Vif should induce the virus to replicate selectively in the inoculated SupT1 cells, thereby protecting the CD4+ T cells of the host. Several molecules show promising anti-Vif activity [8,9,10]. APOBEC3G is expressed by different cell types, such as neuronal cells, astrocytes and macrophages [11]; thus, the pharmacological inhibition of Vif may reduce the formation of HIV reservoirs in the brain and other body areas, while at the same time leaving viral replication in SupT1 cells unaffected. This may prevent the development of pharmacoresistance to Vif inhibitors. In addition, protecting the CD4+ T cells of the host from virus-mediated killing may prevent the development of the chronic immune activation observed in HIV-infected individuals. Another consideration is the potential enhancement of antiviral immunity. Previous studies suggest that preserved IL-21 production correlates with the improved control of viral replication observed in HIV elite controllers [12,13]; therefore, SupT1 cells transfected with an IL-21 expression plasmid may help control viremia in the host. 

Finally, we must consider the possible development of an allogeneic response against the infused SupT1 cells. To become a reservoir for HIV-1, infected cells must survive HIV-1 replication and escape immune recognition. However, SupT1 cells should not escape immune recognition for long; therefore, it seems unlikely that SupT1 cells will become a reservoir for the virus. In addition, an allogeneic response against SupT1 cells may help ensure the eradication of the inoculated cells. In this regard, it should be mentioned that inoculation with tumor cells has been attempted in cancer patients [14,15]. In such cases, the tumor cells are irradiated prior to inoculation and are therefore unable to replicate. A similar protocol could be used to improve the safety of SupT1 cell inoculation. Previous *in vitro* studies show that irradiation of PBMC and T cell lines prior to infection enhances HIV replication [16,17], suggesting that the irradiated SupT1 cells should support optimal HIV replication. In addition, irradiation enhances tumor cell antigenicity recognized by tumor-specific CD8+ T cells [18]. 

## 3. Conclusions

Considering the data available in the literature, the SupT1 cell line seems to be a promising candidate for exploring the possible pacific coexistence between the HIV-1 virus and its host in HIV-susceptible animal models. Such studies may lead to the development of a functional cure for HIV infection. 
